# Applying machine learning technologies to explore students’ learning features and performance prediction

**DOI:** 10.3389/fnins.2022.1018005

**Published:** 2022-12-22

**Authors:** Yu-Sheng Su, Yu-Da Lin, Tai-Quan Liu

**Affiliations:** Department of Computer Science and Engineering, National Taiwan Ocean University, Keelung City, Taiwan

**Keywords:** programming courses, machine learning technologies, learning features, learning performance prediction, algorithms

## Abstract

To understand students’ learning behaviors, this study uses machine learning technologies to analyze the data of interactive learning environments, and then predicts students’ learning outcomes. This study adopted a variety of machine learning classification methods, quizzes, and programming system logs, found that students’ learning characteristics were correlated with their learning performance when they encountered similar programming practice. In this study, we used random forest (RF), support vector machine (SVM), logistic regression (LR), and neural network (NN) algorithms to predict whether students would submit on time for the course. Among them, the NN algorithm showed the best prediction results. Education-related data can be predicted by machine learning techniques, and different machine learning models with different hyperparameters can be used to obtain better results.

## 1. Introduction

With the rapid development of the internet, machine learning is developing at a fast pace. Through the online learning environments, students’ learning logs can be recorded, and a large number of learning characteristics can be obtained. Previous studies applied machine learning algorithms to analyze the system logs to predict students’ learning performance (Romero et al., 2013; Lu et al., 2018). By predicting the performance of at-risk students, teachers are able to intervene with students at an early stage to improve their chances of getting credit for the course (Lu et al., 2018; Huang et al., 2020).

In the field of machine learning, classification, clustering, and regression are the three main algorithms. In recent years, improved algorithms have been proposed that are based on these three algorithms. Therefore, this study extracts students’ learning features from system logs in a programming system to predict their learning performance, and compares the differences in the prediction results of different machine learning algorithms.

This study explores students’ learning features and learning outcomes by using machine learning classification algorithms to conduct an experimental activity with students in a northern college and university. Moreover, the study proposes the following two research questions.

• What is the relationship between learning features and learning performance in programming practices?
• Which learning machine classification algorithms are suitable for predicting students’ learning performance in programming practices?

## 2. Literature review

### 2.1 Learning performance prediction

Predicting the learning outcomes of learners is a common focus of educational data exploration (Baker, 2010). Previous studies used different educational data and characteristics to find the relationship with learning outcomes (Bhardwaj and Pal, 2012; Nahar et al., 2021). For example, Bhardwaj and Pal (2012) used the Naïve Bayes classification algorithm to predict students’ learning outcomes based on the learning characteristics of age, gender, family wealth, residence in a dormitory, and irrelevance to learning achievements. Their experimental results found that these learning characteristics may affect learning performance. However, their results found that the questionnaire survey process may cause unhappiness among students. Nahar et al. (2021) conducted an analytical study to explore semester grades and classroom participation using manually collected data on semester grades, classroom participation, and accompanying tests in required courses. We found that this study was labor-intensive due to manually collecting information on classroom activities. Previous studies (Jong et al., 2007; Ming and Ming, 2012; Lu et al., 2018; Huang et al., 2020) have found that online programming systems record useful student manipulation data. They used the data to predict whether students would have learning difficulties so that they could remedy them in a timely manner. Therefore, we believe that a programming system can save a lot of labor and expense in collecting data and information about course activities.

In this study, we combined classroom performance (e.g., attendances, quizzes, etc.,) and the system logs from the programming system to predict students’ learning outcomes through suitable machine learning classification models.

### 2.2 Machine learning classification algorithms

To understand the relationship between learning characteristics and course performance, Romero and Ventura (2010) conducted a review of educational data exploration techniques. At the same time, they presented many case studies demonstrating that machine learning algorithms are effective in terms of predicting student performance. Al-Radaideh et al. (2006) used the decision tree for educational data analysis, including C4.5, id3, and Naïve Bayes algorithms, but did not achieve the expected results. Osmanbegović et al. (2014) uses information gain, gain ratio, and rank search to remove less influential data to reduce the complexity of models, and found that among tree based algorithms, random forest (RF) performed best. In the study by Huang et al. (2020), the use of logistic regression (LR), support vector machine (SVM), decision tree (DT), RF, and neural network (NN) algorithms was able to predict at-risk students, and the Spearman correlation method was used to explore important features that may affect the classification prediction results. Cardona et al. (2020) mentioned that NN, DT, LR, and SVM algorithms are the most commonly used educational data mining algorithms, and determined the range of each accuracy (ACC).

In reality, it is impossible for all data to be perfect, and there are bound to be students who perform poorly or are simply lazy. With a great deal of temporary effort, luck, and talent before the test, students are likely to achieve excellent results. Past studies (Hooshyar et al., 2020; Huang et al., 2020) have found that ACC is not the most important indicator for evaluation. Instead, it is more important to identify all at-risk students than to correctly distinguish between positive and negative samples when predicting students’ learning outcomes.

In this study, we used the Spearman correlation method for the pre-processing work. Then, we used LR, SVM, RF, and NN algorithms to predict students’ learning outcomes. We used a composite of area under curve- receiver operating characteristic curve (AUC-ROC), ACC, and F1-score as the evaluation metrics.

## 3. The chatbot-assisted programming system

The chatbot-assisted programming system has six modules, namely the user module, the teacher module, the user profile module, the teaching module, the roll call module, and the learning materials module. The user module divides users into the teacher side and the student side. The teacher side can create learning materials through the system for the student side to use, and students can take quizzes and watch activities through the system. The user profile module helps users to manage their own user data, including student number setting and website password query, and uses the command query function to know what commands are available in the system. The teaching module helps the teacher use the teaching data management functions, including uploading learning materials, creating quizzes, creating programming practices, and updating the password for roll call. In the programming practice function, there are two types: the student side and the teacher side. The student side provides topics for practicing hands-on activities using the interface, and the topics are imported into the online judge system to provide students with programming practice activities. The teacher side allows the teacher to view the status of students’ programming exercises, code submissions, and the use of the chatbot-assisted programming system for each activity. The roll call module helps the students to use the chatbot-assisted programming system to take roll call during each activity, and to check the current roll call record. During and after class, the teacher provides the roll call password of the day in the class to confirm the students’ attendance. The user enters “roll call” and then enters the roll call password of the day, and the chatbot-assisted programming system returns a “roll call successful” message after receiving the reply. The learning materials module helps users to view the programming practice, view the learning materials, and take quizzes in class, and the student number setting can be used to confirm users’ identity and check the password used for the week’s classes to facilitate the activities. There are related quiz questions for each week’s class. Enter “#QA” and the chatbot-assisted programming system replies with this week’s questions. After the user clicks on the question, the chatbot-assisted programming system replies to the relevant picture information of the question, and then answers according to the information given by the question, and the user must answer according to the information given by the question.

## 4. Methodology

### 4.1 Participants

The experimental subjects were 64 undergraduate students participating in a C/C++ programming activity at a college in Taiwan. The participants included 53 males and 11 females. Most of the students were 20–21 year-old freshmen with insufficient development and programming experience. The activity was conducted in a general computer classroom, and each student had a computer during the class. The teacher arranged for the students to use a chatbot-assisted programming system to conduct the course.

### 4.2 Learning materials

This course was based on Thinking in C++ 2nd Edn (Eckel and Allison, 2000). The main learning objectives were to provide students with an understanding of C/C++ programming and to promote students’ C/C++ programming skills. Activities included input/output and basic data types, arrays and strings, constructs and linked strings, dynamic planning, and class inheritance. After students had gained a good understanding of the basic concepts, they worked on programming practice.

### 4.3 Procedure

In this study, we planned to participate in the programming experimental activity, which lasted for 7 weeks; the activity was conducted once a week for 6 h each time.

In the first week of the experiment, the teacher introduced the learning objectives of the activity and the operation of the chatbot-assisted programming system to ensure that each student could use and operate it smoothly.

In the following 5 weeks, the teacher conducted a lecture course followed by a hands-on course. The teacher gave quizzes during the lecture courses. The teacher conducted programming practice in the hands-on course. Students were guided by the teacher and submitted their programing practices to the online judge system. If there were any errors or unclear parts of the programming practice, students could use the chatbot-assisted programming system to view detailed explanations of the programming practice for the practical activities to help students learn step-by-step and improve their logical thinking and programming skills.

In the last week, the teacher conducted the final project. The final project was designed to assess students’ learning performance by combining what they had learned in the previous 5 weeks before they were able to answer it. Finally, the logs of the chatbot-assisted programming system and the operation logs of quizzes and programming practice were organized and analyzed.

### 4.4 Instruments

The instruments included quizzes and programming practices.

#### 4.4.1 Quizzes

In the activity, the teacher designs the important concepts into the form of quizzes so that students can learn in stages. Through continuous feedback, students can identify their learning strengths and weaknesses and they need to work on the goals of the course (Burden, 2010). Each week’s activity has corresponding questions, most of which are fill-in-the-blank or short answer questions, and the number of questions in each week’s activity is about 3–10.

#### 4.4.2 Programming practice

In the second week to the sixth week, students were assigned programming practices based on the course they have learned. The programming practices include input/output, basic data types, arrays, strings, structures, linking lists, dynamic programming, recursion, class inheritance, and virtual functions. If students submit their programming practices on time during each week, they obtain a mark for this question. The score of each question is important for obtaining a certificate at the end of the activity. The certificate will be able to prove their learning performance and be recognized by the school.

### 4.5 Data collection and analysis

The data set was obtained from 67 students in the C/C++ programming activity, but three students who participated were excluded due to there isn’t enough data. The remaining 64 students used the learning features from the chatbot-assisted programming system.

In the experimental activity, we used on-time submission as students’ learning performance, and collected data on quizzes and programming practice as the learning features (Huang et al., 2020; Lee et al., 2021). The programming practice recorded students’ system logs every millisecond. From the beginning to the end of experimental activity, there was a total of 360,000 records at the end of the activity. After processing the data, e.g., the number of correct uploads, a total of 8,704 cells were used for learning feature analysis.

#### 4.5.1 Learning feature analysis

To further analyze the learning characteristics of the students in the programming activity, the system logs of the students in the chatbot-assisted programming system were recorded, and the learning characteristics were included as follows: F1: age, F2: gender, F3: correctness rate of quizzes, F4: completion rate of quizzes, F5: number of times to leave the programming practice, F6: number of times to focus on the programming practice function, F7: scrolling down the page, F8: scrolling up the page, F9: number of times the previous button was clicked on the programming practice function, F10: number of times the next button was clicked on the programming practice function, F11: number of times to click the spinner button for the programming practice function, F12: number of keystrokes entered in the programming practice function, F13: number of times the answer was verified as completely correct (AC), F14: number of times the result of answer verification is wrong (WA), F15: compilation error (CE), F16: time limit exceeded (TLE), and F17: on-time submission to the online judge system. The system operation logs were compiled into learning feature codes, and then the feature codes were processed for data cleaning, feature selection, and other pre-processing works, and the data were normalized. Finally, we observed how each model performed when classifying these data.

Among them, F1–F4 were generated by quizzes, while F5–F17 were generated by the programming practice. The above information was obtained by the chatbot-assisted programming system.

#### 4.5.2 Machine learning classification algorithms

We applied the binary classification algorithms, namely RF, SVM, LR, and NN. Educational data mining accounts for a high percentage of the field (Hu et al., 2014; Abu Saa et al., 2019; Cardona et al., 2020). In recent years, there have been many changes and updates based on the above algorithm. For example, RF has the best performance in tree-based algorithms (Osmanbegović et al., 2014), while the radial basis function (RBF) kernel is regarded as the best choice in the application of SVM to educational materials (Agarwal et al., 2012).

##### 4.5.2.1 Support vector machine

Support vector machine (SVM) mainly deals with the binary classification problem (Cortes and Vapnik, 1995), and is still effective when the number of dimensions is larger than the number of samples. The principle is to map the data points in a multi-dimensional space and to find the best hyperplane that can separate two classes with the maximum interval. Its objective function is Eq. 1.


(1) $$\min\left(\left[\frac{1}{n}\,\sum_{i=1}^{n}\text{max}\,\left(0,1\right)-y_{i}\,\left(w^{T}\,x_{i}-b\right)\right]+\lambda \,{||w||}^{2}\right)$$

Where ***x_i_*** is the input vector, ***y_i_*** is the class to which ***x_i_*** belongs (the value is −1 or 1), ***w*** is the normal vector of the hyperplane, ***b*** is the offset between the hyperplanes, and λ is to ensure that ***x_i_*** is located in the correct one of the edge side. Sometimes the samples are not linearly separable, and the data needs to be transferred to a higher-dimensional space for linear separation. At this time, the non-linear decision boundary is processed by the kernel method. In this study, linear kernel, polynomial kernel, and RBF kernel were used.

##### 4.5.2.2 Logistic regression

Logistic regression (LR) is a logit regression model (Strano and Colosimo, 2006). Its principle is similar to that of general linear regression, but LR uses a sigmoid function with the trainable parameter ***w***, which can be used as one of the metrics for selecting a model to determine whether to increase its complexity, as in Eq. 2.


(2) $$h\,\left(x\right)=\frac{1}{1+e^{-w^{T}\,x}}$$

Use ***h***(***x***) as a logarithmic function to form a cost function as formula (3) and find the ***w*** that minimizes the cost function ***J***(***w***).


(3) $$J\,\left(w\right)=\frac{1}{m}\,\sum_{i=1}^{m}\left[y^{i}\,\text{log}\,\left(h\,\left(x^{i}\right)\right)+\left(1-y^{i}\right)\,\left(1-h\,\left(x^{i}\right)\right)\right]$$

The optimization method of the loss function used in this study is L-BFGS (Limited-memory Broyden–Fletcher–Goldfarb–Shanno). BFGS is a non-linear optimization algorithm of the quasi-Newton method (Fletcher, 2013). The curvature update optimizes the loss function through the approximation of the Hessian matrix, while L-BFGS only keeps the records of the previous curvature updates to save storage space. In addition, Scikit-learn provides a coordinate descent optimization algorithm called LIBLINEAR, which finds the minimum value of the function by continuously minimizing along the coordinate direction (Wright, 2015).

##### 4.5.2.3 Random forest

Random forest (RF) generates uncorrelated decision tree clusters through bagging (Golino and Gomes, 2016). Since a single tree is prone to bias or overfitting, it has more accurate results when an ensemble is formed by multiple unconnected trees.

Sklearn uses gini to calculate the importance of each node, and assumes that there are only two child nodes. In the following formula ***ni_j_*** is the importance of the node, ***w_j_*** is the weighted number of samples reaching node j, and ***C_j_*** is the impurity value of node j.


(4) $$n\,i_{j}=w_{j}\,C_{j}-w_{l\,e\,f\,t\,\left(j\right)}\,C_{l\,e\,f\,t\,\left(j\right)}-w_{r\,i\,g\,h\,t\,\left(j\right)}\,C_{r\,i\,g\,h\,t\,\left(j\right)}$$

Then the importance of each feature on the decision tree can be calculated as the following formula. ***RFf_i_*** is the importance of feature i calculated from all trees in the RF, ***normf_ij_*** is the normalized feature importance for i in tree j, and T is the total number of trees.


(5) $$R\,F\,f_{i}=\frac{\sum_{j\in a\,l\,l\,t\,r\,e\,e\,s}n\,o\,r\,m\,f_{i\,j}}{T}$$

##### 4.5.2.4 Neural network

A neural network (NN) is a mathematical model that simulates the function of a biological NN (Cybenko, 1989), and is composed of multiple layers of artificial neurons, consisting of an input layer, an output layer, and several hidden layers.

The nodes of each layer are composed of an input layer, an output layer, and multiple hidden layers. All of them are connected to the nodes of the next layer. Compared with LR, there can be one or more non-linear layers between the input layer and the output layer. NNs learn in artificial neurons, and each iteration reduces the amount of error in prediction by changing the weight parameters. In formula (6), where *x_i_* is the input vector, *w_i_* is the weight of the *x_i_* vector, b is the threshold value, activate is the activation function, typically using sigmoid, hyperbolic tangent, and rectified linear unit (ReLU), etc.


(6) $$\text{Y}=\text{activate}\,\left(\sum_{i=1}^{m}\left(x_{i}\,w_{i}\right)-b\right)$$

#### 4.5.3 Evaluation metrics

Accuracy, F1 score, and AUC are all calculated using the results of the confusion matrix (Hanley and McNeil, 1982). The F1 score, also known as the F1 measure, is the summed average of precision and recall (Van Rijsbergen, 1979). Precision rate, also known as the true positive rate (TPR), is the ratio at which the predicted sample is positive and the actual sample is positive, while the recall rate is the rate at which the predicted sample is also positive in the positive sample. The AUC-ROC is a commonly used metric (Hanley and McNeil, 1982). The ROC curve is formed by setting a threshold value for different false positive rate (FPR) and TPR. It’s setting *x*-axis with FPR and *y*-axis with TPR, and AUC is the summary of the ROC curve. As with random guessing, a larger AUC value indicates a better model classification.

## 5. Results

### 5.1 Relationship between learning features and learning performance

We compared the correlation between each learning feature of the students in the programming practice and the learning outcomes of the programming practice. We used the Pearson product-difference correlation coefficient for correlation analysis.

As shown in Table 1, the International Standard Book Number (ISBN) programming practice only had significant correlation features among the items in the first 4 weeks. After the fifth week, each item had significant correlation features. Further subdividing the association of each feature with the item, F4 significant correlation occurred on particularly simple items, F5 and F6. The programming practice with significant positive correlation was concentrated after the fifth week. F9 and F10 were significantly positively related questions, F13 was a relatively simple programming practice, and F16 was significantly positively correlated in the last week of the programming practice, which may be for testing students’ ability, due to strict time constraints.

**TABLE 1:** Comparison of the accuracy (ACC) of submissions and the significant positive correlations in each week.

Week	Accuracy	Significant
2nd week	0.953	
3rd week	0.859	F4 (0.317*), F13 (0.252*)
3rd week	0.672	
4th week	0.109	
5th week	0.781	F5 (0.285*), F6 (0.292*)
5th week	0.500	F5 (0.257*), F6 (0.298*), F9 (0.252*), F10 (0.272*), F14 (0.297*)
6th week	0.844	F5 (0.321**), F6 (0.324**), F13 (0.814**)
7th week	0.766	F13 (0.279*), F16 (0.251*)

*p < 0.05; **p < 0.01.

### 5.2 Learning machine classification algorithms for predicting learning outcomes

#### 5.2.1 Data selection

The learning features of F1–F16 are the learning feature inputs and F17 is the output of the machine learning model.

#### 5.2.2 Data pre-processing

There are some anomalous values in the input data of F1–F17 (part of the data of F7, F8, and F10), and the average of that field is used to replace them, while there are no missing values in the whole learning behavior feature codes (F1–F17). To avoid amplifying or minimizing the effect of certain features, the input data of F1–F16 are standardized so that all features show Gaussian distribution, which allows the model to converge faster by using data standardization.

Multicollinearity can lead to some problems, such as numerical instability and overfitting in the machine learning process. It also cannot accurately isolate and understand the effects of individual features. In order to filter out highly correlated features, this study used the Spearman’s rank correlation coefficient to identify non-linear relationships between features in the classification model. According to Dancey and Reidy (2017) who suggest that correlation is weak when it is less than 0.4, we use Spearman’s correlation coefficients method and set a threshold value of less than 0.4.

**FIGURE 1:**
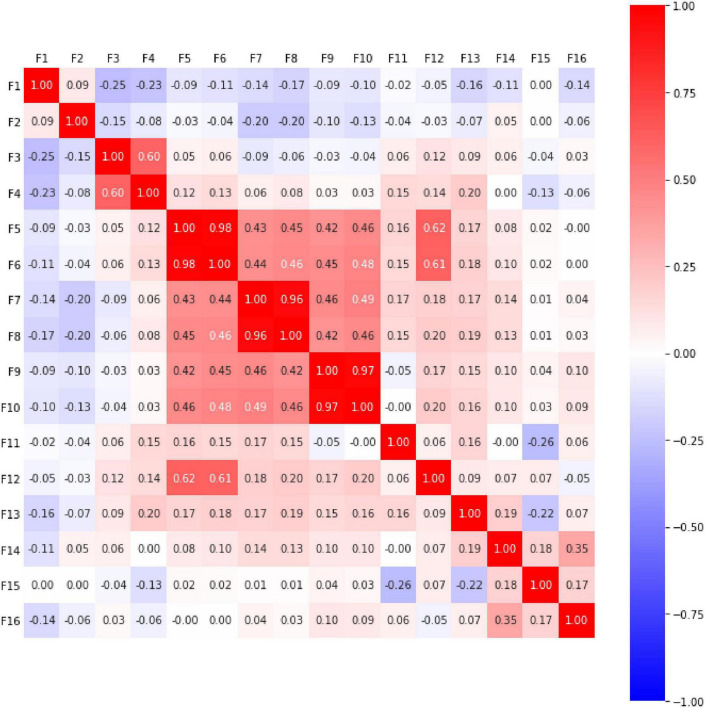
Spearman’s rank correlation coefficients for all features.

The Spearman rank correlation coefficients of all input features are shown in Figure 1. The ρ value is between +1 and −1. When the ρ value is closer to 1 (shown in dark red), it means that the two features have similar trends. When ρ is close to 0 (shown in white), it means that the two features are not related. By setting the threshold value (| Tr| < 0.4), it can be found that in the first and second rows of Figure 1, the ρ value of F1 and F2 in the correlation with each of the other columns is less than 0.4. Until the third row (F3), its ρ value with F4 is 0.6, which is greater than 0.4. We must remove one of F3 (correctness rate of the quizzes) and F4 (Completion rate of quizzes). Because unanswered questions are counted as incorrect, students with low quiz completion rates have low correctness rates. By setting F3 as a feature, the ability of students can be better distinguished. Therefore, we chose F3 as the feature. In F5–F10, 0.98, 0.43, 0.45, 0.42, 0.46 are all greater than 0.4, which means the features from F5–F10 were highly correlated. Of these features, they all leave the current writing area for query and review. Among them, we use F9 as the most representative feature, because the number of times the previous button was clicked can directly reflect that the student is reviewing, and F5 and F6 may just be students taking a break, while F7 and F8 were interfered with by user habits. The ρ value of row F11 is all less than 0.4, while the ρ value of row F12 is greater than 0.4 in F5 and F6, but F5 and F6 have been removed, so F12 continues to be used. The cells of rows F13–F16 are all less than 0.4, so they are also reserved. The final selected input features are F1, F2, F3, F9, F11, F12, F13, F14, F15, and F16.

#### 5.2.3 Model selection

A high value of *C* indicates that the allowable error is too small, which is prone to overfitting; a low value of *C* is prone to underfitting (Hsu et al., 2003). The SVMs were compared in the RBF kernel, linear kernel, and polynomial kernel with different numbers of times (degree, d) to choose different normalization parameters, *C*. The best value of *C* = 1 was found in the RBF kernel.

#### 5.2.4 Model evaluation

The data of the seven exercises of the 5-week internship were used as the training set, and the data of the last week of the program implementation test were used as the test set. F1, F2, F3, F9, F11, F12, F13, F1, F15, and F16 were used as inputs and F17 was used as output, and F1 score, ACC, and AUC were used as the evaluation metrics. The classification prediction results are shown in Table 2.

**TABLE 2:** Model evaluation results.

	ACC	F1	AUC	ACC (Rank)	F1 (Rank)	AUC (Rank)	Overall performance
SVM	0.781	0.863	0.735	2	2	2	**6
LR_liblinear	0.703	0.819	0.597	4	4	4	12
LR_lbgfs	0.703	0.819	0.597	4	4	4	12
RF	0.781	0.857	0.796	2	3	1	**6
NN	0.813	0.880	0.663	1	1	3	*5

*Best overall performance.

**Better overall performance.

## 6. Discussion and conclusion

In this study, we applied machine learning technologies to understand the relationship between student learning features and programming performance. Moreover, we identified appropriate machine learning algorithms for predicting students’ learning performance.

### 6.1 Association between learning features and learning outcomes in programming practice

The Pearson product-moment correlation coefficient was used to correlate students’ learning characteristics with their ability to practice the program and to compare the number of weeks of programming practice problems, correct submission rates, and significant positive correlations. The results showed that the significant correlation characteristics were mostly concentrated in the activities after the fifth week, and students reported higher F5 and F6 in the problems after the fifth week. Since the later activities were often programmed using previous knowledge, students had to look up a large number of online resources or reference books to review what they had learned before they could answer this question. Questions with higher difficulty level showed higher F9 and F10. From this, it can be inferred that the students must read the description of the question repeatedly to clarify and solve the problem. Students could answer this question with logical thinking. Students with lower difficulty problems showed higher F13, thus judging that students will be more active in finding different solutions for easier problems.

In general, students showed similar results for questions of a similar nature, as Hwang et al. (2012) found that student learning behaviors were related to learning performance using educational data mining, and the association between students’ learning characteristic behaviors and learning performance could be explored through educational data mining.

### 6.2 Suitable modeling algorithms for predicting students’ learning outcomes in programming practice

In this study, the classification models the SVM, LR, RF, and NN algorithms, were used to predict conducted with the ACC, F1 score, and AUC as a composite assessment. The NN algorithm performed the best, the SVM, and RF algorithms performed the second and the LR algorithm performed the worst. It is speculated that there are non-linear features in the input space, this result also reflects that SVM performs better in RBF kernel than linear kernel. This result is similar to Ciolacu et al. (2017). By using different machine learning models, namely SVM, LR, DT, NN, and different hyperparameters (regularization, parameters, loss function optimization method, maximum depth of decision number, the NN algorithm hidden layer configuration and regularization parameters, and dropout retention rate) better results can be achieved (Lee et al., 2021). Whether students submitted on time for the day’s activities. The binary classification prediction was the ACC of all the above algorithms is also in the ACC range proposed by Cardona et al. (2020). In addition to trying to use improved algorithms, the reasons for not being able to achieve 100% ACC may also be due to consider students’ individual test preparation habits and other personal factors, such as the research of Nahar et al. (2021) which collected information on other compulsory subjects and analyzed it together.

## 7. Future work

The present findings confirm that the learning features we collected are helpful. This provides a good starting point for learning performance prediction and for improving our proposed system. In recent years, the learning features of online programming systems have been predicted and analyzed using deep learning (Yong et al., 2022). Future works could fruitfully explore this issue further by advanced deep learning algorithms. We will continue to improve our system to be able to collect a wider variety of features.

## Author contributions

All authors contributed equally to the conception of the idea, implementing and analyzing the experimental results, wrote the manuscript, read, and approved the final manuscript.
